# Prognostic Factors and Recurrence in Papillary Thyroid Microcarcinoma

**DOI:** 10.3390/medicina62050981

**Published:** 2026-05-17

**Authors:** Aydan Farzaliyeva, Feride Pınar Altay, Ozlem Turhan Iyidir, Neslihan Bascil Tutuncu

**Affiliations:** 1 Department of Medical Oncology, Faculty of Medicine, Baskent University, 06490 Ankara, Türkiye; 2 Department of Endocrinology and Metabolic Diseases, Ankara Bilkent City Hospital, 06800 Ankara, Türkiye; 3 Department of Endocrinology and Metabolic Diseases, Faculty of Medicine, Baskent University, 06490 Ankara, Türkiye

**Keywords:** Papillary Thyroid Microcarcinoma, prognostic factors, Lymph Node Metastasis, Recurrence, active surveillance, radioactive iodine therapy

## Abstract

*Background and Objectives*: Papillary thyroid microcarcinoma (PTMC) is generally indolent; however, a subset exhibits aggressive features, reflecting biological heterogeneity. In the era of treatment de-escalation and active surveillance, accurate risk stratification is essential. We aimed to evaluate recurrence, identify factors associated with recurrence, determine predictors of lymph node metastasis (LNM) at diagnosis, and assess management strategies at our center. *Materials and Methods:* This retrospective study included 302 patients with PTMC. Associations between clinicopathological variables and outcomes were evaluated using chi-square test, Spearman correlation, and univariate and multivariate logistic regression analyses. *Results*: The cohort included 240 females (79.5%) and 62 males (20.5%), with a median age of 47 years. Total thyroidectomy was performed in 97.7%, and radioactive iodine (RAI) in 64.2%. LNM was identified in 26 patients (8.6%). Recurrence occurred in 4 patients (1.3%), and 98.0% were alive at last follow-up. Recurrence was associated with LNM at diagnosis, higher ATA risk categories, and positive surgical margins (*p* < 0.005). Younger age, larger tumor size, and vascular invasion independently predicted LNM (all *p* < 0.05), while autoimmune thyroiditis was associated with a reduced risk (*p* = 0.020). *Conclusions*: PTMC demonstrates clinically relevant heterogeneity, particularly in patients with LNM. However, given the limited number of recurrence events, recurrence-related findings should be interpreted cautiously and considered exploratory. These findings support risk-adapted management and careful patient selection in the era of treatment de-escalation.

## 1. Introduction

Papillary thyroid carcinoma (PTC) is the most common endocrine malignancy, and its global incidence has been steadily increasing over the past decades, largely driven by advances in diagnostic imaging and enhanced detection of small, subclinical tumors [1,2]. Recent epidemiological analyses confirm that this rising trend continues worldwide, particularly for papillary thyroid microcarcinoma (PTMC), defined as tumors measuring ≤10 mm in diameter [2]. Despite the increasing incidence, PTMC is generally characterized by an indolent clinical course and excellent long-term outcomes, with disease-specific survival rates exceeding 95–98% at 10 years [3,4,5]. However, a subset of patients experiences disease recurrence, highlighting the biological heterogeneity of this entity. Reported recurrence rates in PTMC are typically low, ranging between approximately 3% and 5%, although long-term follow-up studies have demonstrated a gradual increase over time, with recurrence rates reaching approximately 6% and 8% at 20 and 40 years, respectively [6,7,8]. In recent years, management strategies for PTMC have undergone a paradigm shift toward de-escalation, reflecting its generally favorable prognosis. Contemporary guidelines increasingly support less aggressive surgical approaches, including thyroid lobectomy instead of total thyroidectomy in appropriately selected patients, as well as a more selective use of radioactive iodine (RAI) therapy [5,9,10]. Furthermore, active surveillance has emerged as a widely accepted alternative to immediate surgery for low-risk PTMC, with accumulating evidence demonstrating its safety and feasibility in carefully selected cases [10,11]. Nevertheless, a proportion of PTMC cases exhibit more aggressive behavior, including recurrence despite adequate initial treatment or disease progression during active surveillance, underscoring the need for improved risk stratification [6,11,12].

Although various clinicopathological factors have been investigated in relation to recurrence in PTMC, the current evidence remains inconsistent and insufficiently conclusive [11,13,14,15]. In this context, lymph node metastasis (LNM) at the time of diagnosis has emerged as a clinically relevant feature, even in tumors of small size [16,17]. The presence of LNM has important therapeutic implications, as it is associated with the need for more extensive surgical management, including lymph node dissection, and may influence the decision to administer adjuvant RAI therapy. Moreover, patients with LNM are generally not considered suitable candidates for active surveillance under current guideline recommendations.

Importantly, lymph node metastases may be occult at the time of diagnosis and are not always detectable by preoperative imaging modalities, potentially leading to underestimation of disease burden. This limitation raises concerns regarding the inappropriate selection of patients for conservative management strategies, such as active surveillance, in whom clinically relevant nodal disease may remain unrecognized.

Although several clinicopathological factors—including age, sex, tumor size, and extrathyroidal extension (ETE)—have been proposed as predictors of LNM in PTMC [18,19,20], the available evidence remains inconsistent, highlighting the need for further investigation in well-characterized clinical cohorts.

In this study, we aimed to first evaluate recurrence and identify clinicopathological factors associated with recurrence, as well as to determine factors associated with lymph node metastasis (LNM) at the time of diagnosis, and to examine the clinical management and treatment strategies implemented at our center.

## 2. Materials and Methods

This retrospective cohort study was conducted at Başkent University Ankara Hospital and included patients diagnosed with papillary thyroid carcinoma between January 2011 and December 2021. A total of 1800 patients were identified during this period, and those with papillary thyroid microcarcinoma (≤1 cm) were included in the study. Patients younger than 18 years of age were excluded. In addition, patients with incomplete clinicopathological or follow-up data were excluded. Patients with concomitant thyroid malignancies other than papillary thyroid carcinoma, synchronous non-thyroid malignancies, or a history of prior thyroid cancer treatment at another institution were also excluded. Patients who were not regularly followed at our center were excluded to ensure data consistency and reliability. Following the application of the inclusion and exclusion criteria, the final study cohort consisted of 302 patients. We reviewed the medical records, collecting clinicopathological data including age, sex, surgical reports, postoperative RAI administration, and pathological findings.

Patients were followed at 3–6-month intervals during the first year and at 6–12-month intervals thereafter. Follow-up evaluations included serum TSH, thyroglobulin (Tg), and anti-thyroglobulin antibody measurements, along with clinical neck examination and cervical ultrasonography. The analysis included age at diagnosis, sex, tumor size (≤5 mm), multifocality, bilaterality, thyroid capsule invasion (TCI), extrathyroidal extension (ETE), and histopathological variants of papillary thyroid carcinoma. Histopathological variants of papillary thyroid carcinoma were classified according to the 2022 World Health Organization (WHO) classification of thyroid tumors. Tumors were classified as multifocal when two or more tumor foci were present in one or both lobes; in such cases, tumor size was defined based on the maximum diameter of the dominant lesion. Surgical procedures were classified into four groups: total thyroidectomy, total thyroidectomy with ipsilateral lymph node dissection, total thyroidectomy with bilateral lymph node dissection, and lobectomy. In addition, the receipt of postoperative radioactive iodine (RAI) therapy was recorded for each patient.

Tumor staging was performed according to the 8th edition of the American Joint Committee on Cancer (AJCC) TNM classification system, published in 2017, for differentiated thyroid carcinoma [21]. This staging system incorporates tumor size and extent (T), regional lymph node involvement (N), and the presence of distant metastasis (M) to classify disease stage. Furthermore, patients were categorized into four groups based on the 2025 American Thyroid Association (ATA) risk stratification system: low, low–intermediate, intermediate–high, and high risk [10].

Recurrence was defined as the detection of lymph node metastasis (LNM), a recurrent mass, or distant metastasis on neck ultrasonography and/or whole-body scintigraphy after at least 6 months of remission.

### 2.1. Ethical Considerations

This study was approved by the Başkent University Faculty of Medicine Research and Ethics Committee (Project No: KA21/384; approval date: 21 September 2021). In the original ethics committee application, a specific patient inclusion time interval was not explicitly predefined, and the study was approved as a retrospective evaluation of patients with papillary thyroid carcinoma treated at our institution. Following ethics approval, retrospective chart review, data extraction, and verification continued, and consecutive eligible patients diagnosed up to December 2021 were included in the final cohort. Given the non-interventional and anonymized design of the study, the requirement for written informed consent was waived by the Institutional Ethics Committee.

### 2.2. Statistical Analysis

Statistical analyses were performed using IBM SPSS Statistics version 31.0 (IBM Corp., Armonk, NY, USA). The distribution of continuous variables was assessed by examining skewness and kurtosis values, and variables within ±1.96 were considered to follow a normal distribution. Continuous variables were presented as mean ± standard deviation or median (minimum–maximum), while categorical variables were expressed as counts (n) and percentages (%).

Comparisons between groups according to recurrence and lymph node metastasis status were performed using the chi-square test for categorical variables. Spearman correlation analysis was conducted to evaluate the relationships between variables, and the results were reported as correlation coefficients (r) with corresponding two-tailed *p*-values. The strength of correlations was interpreted according to established thresholds: 0.00–0.29 (weak), 0.30–0.69 (moderate), and 0.70–1.00 (strong).

Logistic regression analyses were performed to identify factors associated with lymph node metastasis. Initially, potential predictors were evaluated using univariate logistic regression analysis. Variables that were statistically significant or considered clinically relevant were subsequently included in the multivariate logistic regression model to identify independent predictors. Results were reported as odds ratios (ORs) with 95% confidence intervals (CIs). Model performance was assessed using the Nagelkerke R^2^ coefficient. A *p*-value < 0.05 was considered statistically significant.

## 3. Results

A total of 302 patients diagnosed with micropapillary thyroid carcinoma were included in the analysis. Descriptive findings regarding the demographic, clinical, and pathological characteristics of the patients are presented in Table 1. The median age at diagnosis was 47 years (range: 19–79).

**Figure 1:**
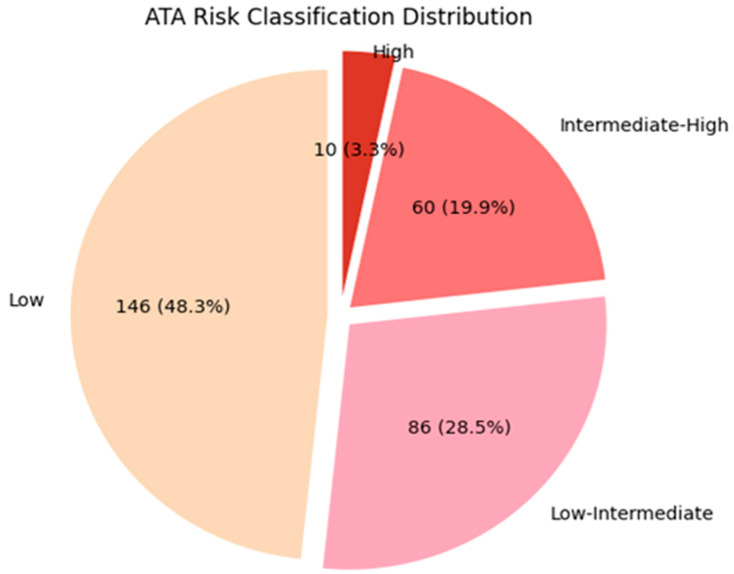
Distribution of patients with micropapillary thyroid carcinoma according to the 2025 ATA risk stratification system.

According to the ATA 2025 risk stratification, 146 patients (48.3%) were classified as low risk, 86 (28.5%) as low–intermediate risk, 60 (19.9%) as intermediate–high risk, and 10 (3.3%) as high risk. Figure 1 illustrates the distribution of ATA 2025 risk categories among patients with micropapillary thyroid carcinoma.

Surgical management consisted predominantly of total thyroidectomy (TT), which was performed in 82.1% of patients, whereas more extensive procedures—including TT with ipsilateral or bilateral lymph node dissection—were less frequent (8.9% and 6.6%, respectively); lobectomy was performed in only 2.3% of cases. Radioactive iodine (RAI) therapy was administered to the majority of patients (64.2%), whereas 35.8% did not receive RAI.

We compared the demographic and clinicopathological characteristics of patients according to RAI therapy status to identify the factors associated with the preferential use of RAI therapy in our center (Table 2).

RAI therapy status was not significantly associated with sex, age, disease stage, histological type, or perineural invasion (all *p* > 0.05). Conversely, bilateral tumor localization and multifocality were significantly more common among patients receiving RAI therapy (both *p* < 0.001).

With respect to tumor size, tumors > 5 mm were significantly more frequent in patients who received RAI therapy (*p* < 0.001). In terms of pathological features, vascular invasion (*p* < 0.001), extrathyroidal extension (*p* < 0.001), and capsular invasion (*p* < 0.001) were significantly more common in the RAI-treated group. Additionally, positive surgical margins were observed at a higher rate among patients who received RAI therapy (*p* = 0.006).

Four patients (1.3%) diagnosed with PTMC developed recurrence during follow-up. At the end of the study, all four patients were alive. Two patients experienced regional recurrence in the form of lymph node metastasis (LNM), one patient developed a recurrent mass at the level of the hyoid bone, and one patient presented with both LNM and distant metastases. The two patients with LNM recurrence were treated with lymph node dissection (LND) followed by radioactive iodine (RAI) therapy and are currently in remission. The patient with recurrence at the hyoid bone level underwent surgical resection followed by RAI therapy and is also being followed in remission.

The patient who developed lung metastases initially received RAI therapy; however, due to the development of RAI resistance, treatment was switched to the tyrosine kinase inhibitor sorafenib, and stereotactic body radiotherapy (SBRT) was administered for brain metastases.

Given the limited number of recurrence events, recurrence-related findings were described descriptively without formal statistical inference. Patients with recurrence appeared more likely to present with lymph node metastasis at diagnosis, positive surgical margins, and higher ATA risk categories (Table 3).

Due to the very small number of recurrence events (1.3%), statistical comparisons should be interpreted with caution. Notably, despite aggressive surgical and RAI management, lymph node metastasis at diagnosis was the most common feature observed among patients with recurrence. Lymph node metastasis in our study was identified in 26 patients (8.6%).

To identify factors predicting lymph node metastasis at diagnosis in micropapillary thyroid carcinoma, we performed a comparative analysis of patients with and without lymph node metastasis to evaluate the associated demographic and clinicopathological characteristics (Table 4).

A statistically significant difference was observed between age groups and the presence of lymph node metastasis (*p* = 0.031), with a higher proportion of patients younger than 45 years in the lymph node metastasis group. In terms of pathological features, vascular invasion was strongly associated with lymph node metastasis (*p* < 0.001). Additionally, tumor size was significantly associated with lymph node metastasis (*p* = 0.023). Among patients without lymph node metastasis, 40.2% had a tumor size of ≤5 mm, compared to only 15.4% in those with lymph node metastasis. Conversely, tumors > 5 mm were more frequent in patients with lymph node metastasis (84.6%).

A statistically significant association was observed between the presence of autoimmune thyroiditis and lymph node metastasis (*p* = 0.027), with autoimmune thyroiditis being less frequent among patients with lymph node metastasis.

In contrast, no statistically significant differences were observed between the groups in terms of sex, tumor laterality, number of tumor foci, histological type, extrathyroidal extension, capsular invasion, surgical margin status, or perineural invasion (*p* > 0.05).

Recurrence was observed significantly more frequently in patients with lymph node metastasis (*p* < 0.001).

**Figure 2:**
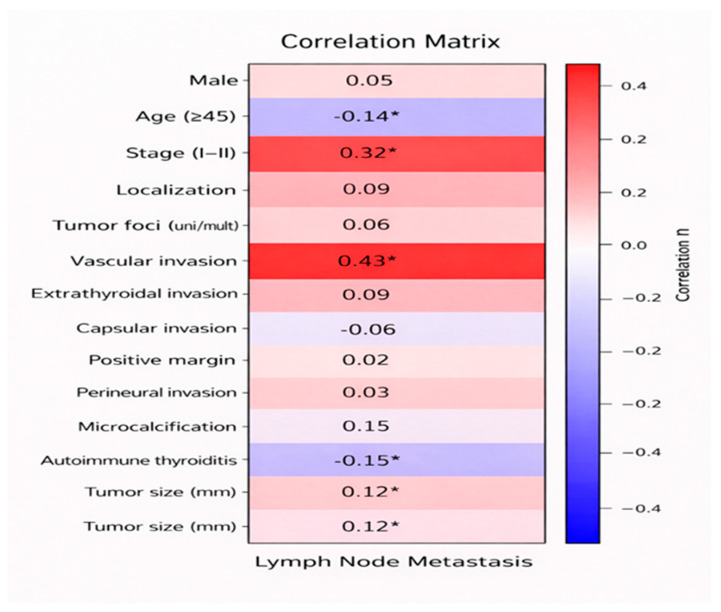
Heatmap showing the correlations between lymph node metastasis and demographic, clinical, and pathological variables in patients with micropapillary thyroid carcinoma. * *p*-value < 0.05.

In addition, correlation analysis was performed to assess the relationships between lymph node metastasis and demographic, clinical, and pathological variables in patients with micropapillary thyroid carcinoma, and the results are shown in Table 5 and Figure 2.

### Predictors of Lymph Node Metastasis in Papillary Thyroid Microcarcinoma: Univariate and Multivariate Logistic Regression Analysis

To identify potential predictors of lymph node metastasis, univariate logistic regression analysis was initially performed. In this analysis, demographic and clinical variables considered as potential prognostic factors were individually entered into the model to evaluate their associations with lymph node metastasis. Variables found to be statistically significant in the univariate analysis, along with those deemed clinically relevant, were subsequently included in the multivariate logistic regression model. This approach allowed for adjustment of potential confounding effects and identification of independent predictors of lymph node metastasis. The results of the univariate and multivariate logistic regression analyses are presented in Table 6.

In the univariate logistic regression analysis, age, disease stage, vascular invasion, presence of autoimmune thyroiditis, and tumor size were found to be significantly associated with lymph node metastasis (*p* < 0.05). Odds ratios (ORs) indicated that values greater than 1 were associated with increased risk, whereas values less than 1 suggested a protective effect. Accordingly, patients aged ≥ 45 years had a lower likelihood of lymph node metastasis (OR = 0.373, 95% CI: 0.160–0.865, *p* = 0.022). Similarly, the presence of vascular invasion increased the risk of metastasis by nearly 19-fold (OR = 18.857, 95% CI: 7.192–49.444, *p* < 0.001). The presence of autoimmune thyroiditis was associated with a reduced risk of metastasis (OR = 0.299, 95% CI: 0.108–0.830, *p* = 0.020). In addition, each 1 mm increase in tumor size was associated with an approximately 1.2-fold increase in the likelihood of metastasis (OR = 1.218, 95% CI: 1.019–1.455, *p* = 0.025).

In the multivariate logistic regression analysis, age, tumor size, and vascular invasion remained independent predictors of lymph node metastasis. Patients aged ≥ 45 years had a lower risk of metastasis (OR = 0.292, 95% CI: 0.087–0.984, *p* = 0.047). Vascular invasion was identified as a strong independent predictor, increasing the likelihood of metastasis by approximately 11.7-fold (OR = 11.672, 95% CI: 3.754–36.293, *p* < 0.001). Tumor size (mm) was identified as a statistically significant factor in multivariable analysis (OR: 1.20, 95% CI: 1.02–1.45, *p* = 0.030). In contrast, autoimmune thyroiditis (*p* = 0.101) lost statistical significance in the multivariate model and was not identified as an independent predictor. The model demonstrated a Nagelkerke R^2^ of 0.369.

## 4. Discussion

In the present study, we provide a comprehensive evaluation of clinicopathological characteristics in patients with papillary thyroid microcarcinoma (PTMC), describe recurrence patterns, and investigate factors associated with lymph node metastasis within a real-world clinical setting, including the treatment strategies applied at our center. In the context of the ongoing paradigm shift toward treatment de-escalation and active surveillance, identifying patients at risk for more aggressive disease—particularly those with lymph node metastasis (LNM)—remains of critical importance.

Consistent with the overall indolent nature of PTMC, the observed recurrence rate in our cohort was notably low (1.3%), appearing lower than that reported in most contemporary series, where recurrence rates typically range from approximately 3% to 6% following surgical management [9,13,14,22].

This finding may be partly explained by the higher likelihood of early-stage disease at diagnosis, reflecting the increasing use of sensitive diagnostic modalities and consequent earlier detection before metastatic spread. In addition, relatively more extensive initial management strategies applied in our population may have contributed to the reduced recurrence risk. Specifically, total thyroidectomy was performed in 97.7% of patients, while only a small proportion (*n* = 7) underwent lobectomy. Current international guidelines, such as the 2025 ATA framework and the 2025 KTA update, increasingly advocate for active surveillance or less extensive surgical interventions—specifically favoring thyroid lobectomy over total thyroidectomy—as the primary surgical approach for low-risk PTMC [9,10]. These updates emphasize that even in cases of multifocality, a more limited surgical approach (such as thyroid lobectomy) is oncologically safe, provided there is no clinical evidence of lymph node metastasis or gross extrathyroidal extension. This global shift toward therapeutic de-escalation is further supported by recent long-term data showing that lobectomy often provides equivalent oncological safety to more extensive procedures in well-selected patients [23]. However, it should be noted that our study covered the period between 2011 and 2021, during which the long-term outcomes of active surveillance strategies were not yet clearly established, and more aggressive surgical approaches continued to be widely adopted in routine clinical practice. In particular, total thyroidectomy was more frequently preferred in the presence of intermediate- and high-risk clinicopathological features, including multifocality, bilateral disease, lymph node involvement, and extrathyroidal extension. İn addition, some authors continue to advocate total thyroidectomy due to its association with lower recurrence rates and its utility in postoperative surveillance through thyroglobulin monitoring and radioactive iodine (RAI) imaging [24].

In addition, during the same period, lymph node dissection was performed in 47 patients (15.6%), including 27 patients (8.9%) who underwent total thyroidectomy with ipsilateral lymph node dissection and 20 patients (6.6%) who underwent total thyroidectomy with bilateral lymph node dissection. According to the prevailing surgical practices of that era and the more aggressive management approaches commonly adopted during the study period, lymph node dissection was more frequently performed in patients with clinically suspected nodal involvement and/or intermediate- and high-risk clinicopathological features, including multifocality, bilateral disease, and extrathyroidal extension. Due to the retrospective nature of the study, a consistent subclassification into central versus lateral neck dissection was not available for all patients.

The second pillar of our management strategy, the clinical application of adjuvant RAI therapy, also warrants dedicated consideration in light of current oncological outcomes. In our cohort, a significant proportion of patients (64.2%) received RAI, a decision-making process predominantly shaped by the risk-adapted paradigms of the ATA 2015 era. Notably, nearly 41% of our RAI-treated population consisted of low- or low–intermediate-risk individuals who might be managed by selective observation under the newly published ATA 2025 guidelines [10].

Under the 2015 framework, radioactive iodine remnant ablation (RRA) was frequently utilized even in these low-risk patients to facilitate follow-up by “zeroing” thyroglobulin levels, regardless of minor aggressive features. However, the ATA 2025 update represents a clear shift toward therapeutic de-escalation, specifically by recommending against routine remnant ablation for low-risk PTMC and no longer considering small (<1 cm) bilateral multifocal and unilateral multifocal disease as standalone indications for RAI [5]. In addition, the updated guidelines emphasize a more individualized, response-adapted management strategy, whereby the decision to administer RAI is deferred and guided by dynamic risk stratification based on post-operative findings, including serum thyroglobulin levels and neck ultrasonography. This shift reflects a move away from the traditional pathology-driven approach, used in our center, toward a more selective use of RAI, particularly in low- to intermediate-risk patients, in whom routine administration is no longer justified in the absence of adverse features. Furthermore, the ATA 2025 framework de-emphasizes the role of RAI in routine follow-up, acknowledging that effective surveillance can be achieved without remnant ablation, thereby reducing unnecessary treatment-related morbidity without compromising long-term oncological outcomes [10].

While robust evidence does not support routine RAI use in low-risk PTMC, selected patients within the low–intermediate risk spectrum—particularly those with unilateral multifocality or bilateral multifocal disease <1 cm, who are now classified within the low–intermediate risk group according to the ATA 2025 risk stratification—may still derive benefit from adjuvant RAI due to a higher likelihood of occult microscopic disease. Notably, even when confined to a single lobe, multifocality has been associated with more aggressive clinicopathological features compared to unifocal disease, including a higher tumor burden and increased rates of lymph node metastasis, suggesting a greater extent of subclinical disease [25].

In a recent study conducted by Bilgic et al. evaluating the impact of radioactive iodine (RAI) therapy in patients with low-risk papillary thyroid carcinoma, the RAI-treated group demonstrated a significantly higher proportion of bilateral (30% vs. 9%) and multifocal (33% vs. 7%) disease compared to those managed without adjuvant therapy (*p* < 0.001), indicating that patients with a greater intrathyroidal tumor burden were more likely to receive RAI. Despite this higher-risk profile, the RAI group exhibited lower recurrence rates (1.0% vs. 5.8%) and higher rates of no evidence of disease (99.0% vs. 94.2%), supporting the potential role of RAI in improving disease control. In this context, particularly in patients with multifocal papillary thyroid microcarcinoma, adjuvant RAI may provide additional benefit by targeting residual microscopic foci and potentially reducing the risk of recurrence [26].

Similarly, a large-scale meta-analysis reported that multifocal papillary thyroid carcinoma is associated with an increased risk of recurrence compared to unifocal disease, likely reflecting a higher burden of microscopic disease [27].

In patients with papillary thyroid microcarcinoma (≤1 cm), multifocality has been associated with an increased risk of recurrence (HR ≈ 1.81), indicating that even subcentimeter multifocal disease may carry clinical relevance. However, this effect appears to be dependent on tumor burden, as a significant increase in recurrence risk is observed primarily in patients with ≥3 tumor foci, while two foci alone do not confer a statistically significant risk [27].

Taken together, these findings provide a biological and clinical rationale for considering adjuvant RAI in selected patients with multifocal disease, particularly in those with unilateral or bilateral multifocality with tumor size ≤1 cm, who are now classified within the low–intermediate risk group according to the ATA 2025 risk stratification.

While our adjuvant RAİ strategy differs from contemporary de-escalation trends, our findings suggest that the remarkably low recurrence rates observed in our cohort may, at least in part, be attributable to the proactive use of RAI (particularly in patients with multifocal disease within the low–intermediate risk group)*,* which may have served as a critical safety net by effectively neutralizing occult microscopic disease and thereby contributing to superior long-term disease control.

Given the low number of recurrence events, recurrence-related findings were evaluated descriptively. Patients who developed recurrence tended to present with lymph node metastasis (LNM) at diagnosis, higher ATA risk categories (intermediate–high or high risk), and positive surgical margins. Among these, LNM appeared to be the most prominent feature in patients with recurrence; however, these observations should be interpreted as exploratory rather than definitive. This finding is consistent with previous studies demonstrating that the presence of lymph node metastasis at diagnosis is associated with an increased risk of recurrence in PTMC [16,18,28].

Notably, all patients with LNM in our cohort underwent total thyroidectomy and lymph node dissection, followed by adjuvant RAI therapy. Despite this relatively aggressive management, the persistence of recurrence risk in this subgroup underscores the need for closer surveillance and risk-adapted follow-up strategies in patients presenting with nodal disease at diagnosis, which further prompted us to perform an analysis of factors predicting lymph node metastasis in PTMC.

In the analysis of predictive factors associated with LNM, younger age (<45 years), tumor size > 5 mm, and the presence of vascular invasion were identified as significant predictors in PTMC.

Age emerged as an important determinant of lymph node metastasis in our cohort, with younger patients (<45 years) demonstrating a significantly higher risk of nodal involvement. This inverse relationship between age and LNM risk has been consistently reported in recent studies, although there is no universally accepted age cut-off. Various thresholds have been proposed in the contemporary literature, including 40, 45, and 55 years, reflecting heterogeneity across study populations [20,29,30].

Despite this variability, the overall pattern remains consistent: younger patients tend to present with a higher incidence of LNM and more extensive nodal disease compared to older individuals.

Several recent analyses have also evaluated age as a continuous variable, demonstrating a gradual decline in LNM risk with increasing age.

For example, Liu et al. showed an inverse association between age and nodal metastasis risk in PTMC using multivariable modeling approaches [19]. More advanced modeling techniques, including nonlinear analyses, have further supported this relationship, suggesting a plateau in risk reduction at older ages. The biological basis of this association remains incompletely understood; however, differences in tumor biology, including gene expression profiles and the accumulation of molecular alterations, have been proposed as potential explanations.

In addition, data from active surveillance cohorts indicate that younger age may also be associated with a higher likelihood of disease progression, further supporting its clinical relevance in PTMC management [31,32].

From a clinical perspective, these findings underscore the importance of incorporating age into preoperative risk assessment. A more detailed evaluation of nodal status may be warranted in younger patients, potentially influencing the extent of surgery and follow-up strategies. For instance, younger patients may derive greater benefit from central lymph node dissection, whereas a more conservative approach, such as active surveillance, may be more appropriate in older individuals. However, given the retrospective nature of our study these observations should be interpreted with caution and validated in larger prospective cohorts.

Tumor size also emerged as a significant predictor of lymph node metastasis in our cohort, with lesions >5 mm demonstrating a higher risk of nodal involvement.

The literature reports heterogeneous findings on this issue, with different studies proposing various tumor size cut-offs. While some studies have suggested a 5 mm threshold, others have reported alternative cut-offs such as 6 mm, 7 mm, or 8.5 mm, indicating that no single universally accepted value has yet been established [18,28,33,34].

These findings highlight that, although a universally accepted cut-off is lacking, there is consistent evidence supporting a size-dependent increase in metastatic potential.

Beyond its association with nodal disease, tumor size also plays a central role in determining eligibility for AS. Current data suggest that smaller tumors are less likely to exhibit progression, whereas relatively larger PTMC lesions may carry a higher risk of growth or nodal involvement during follow-up. In particular, a tumor size of ≥6 mm has been reported as a predictor of progression, and growth activity appears to be more pronounced in the 6–9 mm range [5,32].

Even modest increases above 5 mm may reflect a shift toward a more aggressive phenotype, influencing both the likelihood of lymph node metastasis and the risk of progression under active surveillance [5,32]. Therefore, tumor size may be considered as one of several factors in individualized risk stratification when guiding surgical decision-making and selecting patients for AS.

Vascular invasion was also identified as a significant predictor of lymph node metastasis in our cohort, reflecting a more aggressive tumor phenotype. This observation aligns with emerging literature indicating that lymphovascular invasion is associated with nodal metastasis in papillary thyroid microcarcinoma and may represent a marker of tumor invasiveness [12,15,16,17]. Although PTMC is generally considered an indolent disease, the presence of vascular invasion appears to define a higher-risk subgroup with greater metastatic potential. This is particularly relevant in the context of active surveillance (AS), where identifying patients with low-risk disease is essential. Current evidence suggests that patients harboring aggressive pathological features, including vascular invasion, may be less suitable candidates for AS, because these features are associated with a higher likelihood of occult metastatic disease and disease progression. In a recent prospective cohort of patients with PTMC undergoing AS, the overall progression rate was low, but still ranged from approximately 3% to 7% over 2 to 7 years of follow-up, and new lymph node metastasis was one of the key progression events [35]. More importantly, a 2023 study reported that intratumoral vascularity was associated with new lymph node metastasis with a hazard ratio of 5.0, while the risk of tumor progression was higher in patients with this feature compared with those without it (14% vs. 6%) [36]. Similarly, recent pathological data have shown that lymphovascular invasion in PTMC is significantly associated with central nodal metastasis, supporting the concept that microscopic invasive features may identify a biologically more aggressive subgroup [28,35].

Consequently, this pathological feature may have important implications for risk stratification, surveillance intensity, and the selection of patients who might benefit from closer follow-up and more individualized management.

In our study, the presence of autoimmune thyroiditis appeared to have a protective effect in papillary thyroid microcarcinoma, suggesting a less aggressive pathological profile. This finding is consistent with the literature indicating that Hashimoto thyroiditis may exert a protective effect in PTMC by promoting a chronic lymphocytic inflammatory milieu that enhances antitumor immune surveillance and may limit tumor aggressiveness. AITD is characterized by increased lymphocyte and macrophage infiltration as well as the presence of autoimmune antibodies, and this inflammatory environment may lead to follicular destruction and the formation of a fibrotic barrier around the tumor, thereby potentially restricting tumor spread, progression, and metastasis. In addition, Fas-mediated apoptosis associated with AITD has been proposed as another mechanism contributing to improved prognosis in PTC. Recent reports have suggested that papillary thyroid carcinoma associated with Hashimoto’s thyroiditis tends to present with smaller tumor size, less aggressive behavior, and a lower frequency of lymph node invasion, while lymphocytic infiltration may also facilitate earlier detection of low-volume disease. Although this association remains debated and may partly reflect detection bias, the available evidence supports the concept that concomitant autoimmune thyroiditis identifies a distinct biological subgroup of PTMC with potentially more favorable pathological characteristics [37,38,39,40].

This study has several limitations. First, its retrospective and single-center design may restrict the generalizability of the findings. Second, while the cohort size was adequate, the limited number of recurrence events—reflecting the indolent course of PTMC—limited robust statistical analysis for recurrence and requires cautious interpretation. BRAF mutation status was not available for most patients and was therefore not included in the analysis. Another limitation of this study is the absence of a patient group managed with active surveillance, reflecting the more traditional management approach adopted at our center. However, it should be noted that the primary aim of this study was not to evaluate the outcomes of active surveillance but rather to assess clinicopathological characteristics and factors associated with lymph node metastasis. In addition, lymph node dissection was not performed in all patients, which may have resulted in an underestimation of the true incidence of lymph node metastasis. Furthermore, due to the retrospective nature of the study, complete and standardized postoperative complication data and detailed subclassification of lymph node dissection procedures were not consistently available for all patients.

Finally, our study provides meaningful insights into recurrence patterns and the clinicopathological determinants of prognosis—particularly in patients with lymph node metastasis—and, together with existing literature, contributes to the evolving understanding of risk stratification in PTMC in the era of treatment de-escalation.

In conclusion, our findings highlight that papillary thyroid microcarcinoma, although generally considered an indolent disease, may exhibit clinically relevant heterogeneity, particularly in patients with lymph node metastasis. Despite the low overall recurrence rate observed in our cohort, the presence of lymph node metastasis at diagnosis appeared to be associated with an increased risk of recurrence, emphasizing the need for closer surveillance and individualized follow-up strategies in this subgroup.

Younger age, larger tumor size, and the presence of vascular invasion were identified as significant predictors of lymph node metastasis, underscoring their potential role in risk stratification. These findings support a risk-adapted approach to management, the importance of which becomes even more pronounced in the current era of treatment de-escalation and active surveillance in PTMC. Future prospective studies are warranted to further validate these findings and to optimize patient selection for individualized treatment strategies, particularly in the context of active surveillance.

## 5. Conclusions

PTMC demonstrates clinically relevant heterogeneity, particularly in patients with LNM. However, given the limited number of recurrence events, recurrence-related findings should be interpreted cautiously and considered exploratory. These findings highlight the importance of risk-adapted management and refined patient selection, which is increasingly critical in the current era of treatment de-escalation and active surveillance. Future prospective studies are warranted to further validate these findings and optimize individualized treatment strategies.

## Figures and Tables

**Table 1:** Baseline Characteristics of Patients with Micropapillary Thyroid Carcinoma.

Variable	Group	N (%)
Sex	Female	240 (79.5)
	Male	62 (20.5)
Age at diagnosis, years	<45	131 (43.4)
	≥45	171 (56.6)
TNM stage	I	294 (97.4)
	II	8 (2.6)
Laterality	Unilateral	223 (73.8)
	Bilateral	79 (26.2)
Tumor focality	Unifocal	180 (59.6)
	Multifocal	122 (40.4)
Tumor size (mm)	≤5	115 (38.1)
	>5	187 (61.9)
Histologic type	Classic	222 (73.5)
	Follicular	46 (15.2)
	Oncocytic	14 (4.6)
	Other	20 (6.6)
Lymph node metastasis	No	276 (91.4)
	Yes	26 (8.6)
Vascular invasion	No	278 (92.1)
	Yes	24 (7.9)
Extrathyroidal extension	No	270 (89.4)
	Yes	32 (10.6)
Surgical margin status	Negative	284 (94.0)
	Positive	18 (6.0)
Coexisting autoimmune thyroiditis *	No	146 (54.1)
	Yes	124 (45.9)
Capsule invasion	No	245 (81.1)
	Yes	57 (18.9)
Perineural invasion	No	298 (98.7)
	Yes	4 (1.3)
Surgery type	TT TT + ipsilateral LND TT + bilateral LND Lobectomy	248 (82.1) 27 (8.9) 20 (6.6) 7 (2.3)
Radioactive iodine therapy	No	108 (35.8)
	Yes	194 (64.2)
Lymph node dissection	No Yes	255 (84.4) 47 (15.6)
Recurrence	No	298 (98.7)
	Yes	4 (1.3)
Mortality	Alive	296 (98.0)
	Ex	6 (2.0)

Abbreviations: TT, total thyroidectomy; LND, lymph node dissection; TNM, Tumor–Node–Metastasis staging system. * Data on autoimmune thyroiditis were available for 270 patients; data were missing for 32 patients.

**Table 2:** Comparison of demographic, clinical, and pathological characteristics between patients who received and those who did not receive RAI therapy in micropapillary thyroid carcinoma.

Variable	Group	RAİ		
		−	+	
		N (%)	N (%)	*p* Value
Sex	Female	85 (78.7)	155 (79.9)	0.922
	Male	23 (21.3)	39 (20.1)	
Age at diagnosis, years	<45	39 (36.1)	92 (47.4)	0.075
	≥45	69 (63.9)	102 (52.6)	
TNM stage	I	108 (100)	186 (95.9)	0.078
	II	0 (0.0)	8 (4.1)	
Laterality	Unilateral	96 (88.9)	127 (65.5)	<0.001 *
	Bilateral	12 (11.1)	67 (34.5)	
Tumor foci number	Unifocal	86 (79.6)	94 (48.5)	<0.001 *
	Multifocal	22 (20.4)	100 (51.5)	
Tumor size (mm)	≤5	63 (58.3)	52 (26.8)	<0.001 *
	>5	45 (41.7)	142 (73.2)	
Histological type	Classic	84 (77.8)	138 (71.1)	0.427
	Follicular	15 (13.9)	31 (16.0)	
	Oncocytic	5 (4.6)	9 (4.6)	
	Other	4 (3.7)	16 (8.2)	
Vascular invasion	No	108 (100)	170 (87.6)	<0.001 *
	Yes	0 (0.0)	24 (12.4)	
Extrathyroidal extension	No	105 (97.2)	165 (85.1)	<0.001 *
	Yes	3 (2.8)	29 (14.9)	
Capsule invasion	No	101 (93.5)	144 (74.2)	<0.001 *
	Yes	7 (6.5)	50 (25.8)	
Surgical margin status	Negative	107 (99.1)	177 (91.2)	0.006 *
	Positive	1 (0.9)	17 (8.8)	
Perineural invasion	No	108 (100)	190 (97.9)	0.328
	Yes	0 (0.0)	4 (2.1)	

Abbreviations: RAI, radioactive iodine; TNM, Tumor–Node–Metastasis staging system. * *p*-value < 0.05 was considered statistically significant. The chi-square (χ^2^) test was applied to analyze categorical variables.

**Table 3:** Comparison of Demographic, Clinical, and Pathological Characteristics According to Recurrence Status in Patients with Papillary Thyroid Microcarcinoma.

Variable	Group	Recurrence		
		+	−	*p* Value
Sex	Female	2 (50)	239 (80.2)	0.188
	Male	2 (50)	59 (19.2)	
Age at diagnosis, years	<45	3 (75)	130 (43.6)	0.32
	≥45	1 (25)	170 (56.4)	
Laterality	Unilateral	2 (50)	221 (74.1)	0.28
	Bilateral	2 (50)	77 (25.9)	
Tumor focality	Unifocal	2 (50)	178 (59.7)	0.65
	Multifocal	2 (50)	120 (40.3)	
Tumor size (mm)	≤5	3 (75)	112 (37.5)	0.972
	>5	1 (25)	186 (62.5)	
Histologic type	Classic	1 (25)	221 (74.1)	0.83
	Follicular	2 (50)	44 (14.7)	
	Oncocytic	0 (0)	14 (4.7)	
	Other	1(25)	19 (6.5)	
Vascular invasion	No	4 (100)	274 (91.9)	0.999
	Yes	0	24 (8.1)	
Extrathyroidal extension	No	3 (75)	267 (89.6)	0.999
	Yes	1 (25)	31 (10.4)	
Capsule invasion	No	3 (75)	242 (81.2)	0.5
	Yes	1 (25)	56 (18.8)	
Surgical margin status	Negative	2 (50)	282 (94.6)	0.008
	Positive	2 (50)	16 (5.4)	
Perineural invasion	No	4 (100)	294 (98.6)	0.999
	Yes	0	4 (1.4)	
Lymph node metastasis	No Yes	1 (25) 3 (75)	275 (92.3) 23 (7.7)	<0.001
ATA risk group	Low and Low-İntermediate İntermediate-high and High	0 (0) 4 (100)	232(77.8) 66 (22.2)	0.001
TNM stage	I II	3 (75) 1 (25)	291 (97.6) 7 (2.4)	0.12
Surgery type	TT TT + ipsilateral LND TT + bilateral LND Lobectomy	1 (25) 1 (25) 2 (50) 0 (0)	247(82.9) 26 (8.7) 18 (6) 7 (2.4)	0.012
Radioactive iodine therapy	No	0 (0)	194 (65.1)	0.73
	Yes	4 (100)	104 (34.9)	

Abbreviations: LNM, lymph node metastasis; ATA, American Thyroid Association; TNM, Tumor–Node–Metastasis staging system; TT, total thyroidectomy; LND, lymph node dissection; Note: Data regarding underlying autoimmune thyroiditis status in patients with recurrence were unavailable. Due to low number of recurrence events, statistical comparisons should be interpreted with caution.

**Table 4:** Comparison of Demographic, Clinical, and Pathological Characteristics According to the Presence of Lymph Node Metastasis in Patients with Papillary Thyroid Microcarcinoma.

Variable	Group	LNM		
		−	+	*p* Value
Sex	Female	221 (80.1)	19 (73.1)	0.555
	Male	55 (19.9)	7 (26.9)	
Age at diagnosis, years	<45	114 (41.3)	17 (65.4)	0.031 *
	≥45	162 (58.7)	9 (34.6)	
Laterality	Unilateral	207 (75.0)	16 (61.5)	0.208
	Bilateral	69 (25.0)	10 (38.5)	
Tumor focality	Unifocal	167 (60.5)	13 (50.0)	0.404
	Multifocal	109 (39.5)	13 (50.0)	
Tumor size (mm)	≤5	111 (40.2)	4 (15.4)	0.023 *
	>5	165 (59.8)	22 (84.6)	
Histologic type	Classic	206 (74.6)	16 (61.5)	0.427
	Follicular	40 (14.5)	6 (23.1)	
	Oncocytic	13 (4.7)	1 (3.8)	
	Other	17 (6.2)	3 (11.5)	
Vascular invasion	No	264 (95.7)	14 (53.8)	<0.001 *
	Yes	12 (4.3)	12 (46.2)	
Extrathyroidal extension	No	249 (90.2)	21 (80.8)	0.245
	Yes	27 (9.8)	5 (19.2)	
Capsule invasion	No	222 (80.4)	23 (88.5)	0.461
	Yes	54 (19.6)	3 (11.5)	
Surgical margin status	Negative	260 (94.2)	24 (92.3)	1.000
	Positive	16 (5.8)	2 (7.7)	
Perineural invasion	No	273 (98.9)	25 (96.2)	0.780
	Yes	3 (1.1)	1 (3.8)	
Coexisting autoimmune thyroiditis	No	128 (51.8)	18 (78.3)	0.027 *
	Yes	119 (48.2)	5 (21.7)	
Recurrence	No	275 (99.6)	23 (88.5)	<0.001 *
	Yes	1 (0.4)	3 (11.5)	

Abbreviations: LNM, lymph node metastasis. * A *p*-value of <0.05 was considered statistically significant. The chi-square (χ^2^) test was applied for the analysis of categorical variables.

**Table 5:** Correlation Analysis of Factors Associated with Lymph Node Metastasis in Papillary Thyroid Microcarcinoma.

Variable	Presence of Lymph Node Metastasis r	*p* Value
Sex (Male)	0.049	0.400
Age at diagnosis (≥45 years)	−0.136 *	0.018
TNM stage (II)	0.317 *	<0.001
Localizatıon (Laterality)	0.086	0.136
Number of tumor foci (Uni/Multifocal)	0.060	0.298
Vascular invasion presence	0.434 *	<0.001
Extrathyroidal invasion	0.086	0.135
Capsule invasion	−0.058	0.319
Positive surgical margin	0.022	0.698
Perineural invasion	0.068	0.241
Microcalcification	−0.034	0.554
Coexisting autoimmune thyroiditis	−0.148 *	0.015
Tumor size (>5 mm)	0.122 *	0.033

Abbreviations: TNM, Tumor–Node–Metastasis staging system. r: Spearman correlation coefficient. * A *p*-value of <0.05 was considered statistically significant (*p* < 0.05).

**Table 6:** Univariate and Multivariate Logistic Regression Analysis of Factors Predicting Lymph Node Metastasis in Patients with Micropapillary Thyroid Carcinoma.

Variable	Univariate			Multivariate		
	OR	95% CI	*p*	OR	95% CI	*p* Value
Age at diagnosis (≥45)	0.373	0.16–0.86	0.022 *	0.292	0.08–0.98	0.047 *
Vascular invasion	18.857	7.19–49.44	<0.001 *	11.672	3.75–36.29	<0.001 *
Autoimmune thyroiditis	0.299	0.11–0.83	0.020 *	0.369	0.11–1.22	0.101
Tumor size (mm)	1.218	1.01–1.45	0.025 *	1.20	1.02–1.45	0.030 *

Abbreviations: OR: odds ratio; CI: confidence interval. The results of the logistic regression analysis are presented as ORs with 95% CIs. * A *p*-value < 0.05 was considered statistically significant. The explanatory power of the model was indicated by a Nagelkerke R^2^ of 0.369.

## Data Availability

The data presented in this study are available on reasonable request from the corresponding author. The data are not publicly available due to institutional privacy regulations and patient confidentiality.

## Abbreviations

The following abbreviations are used in this manuscript:

PTC	Papillary Thyroid Carcinoma
PTMC	Papillary Thyroid Microcarcinoma
LNM	Lymph Node Metastasis
RAI	Radioactive Iodine
ETE	Extrathyroidal Extension
TCI	Thyroid Capsule Invasion
ATA	American Thyroid Association
AJCC	American Joint Committee on Cancer
TNM	Tumor, Node, Metastasis
Tg	Thyroglobulin
TSH	Thyroid-Stimulating Hormone
LND	Lymph Node Dissection
OR	Odds Ratio
CI	Confidence Interval
